# Human Medulloblastoma Cell Lines: Investigating on Cancer Stem Cell-Like Phenotype

**DOI:** 10.3390/cancers12010226

**Published:** 2020-01-17

**Authors:** Arianna Casciati, Mirella Tanori, Rémi Manczak, Sofiane Saada, Barbara Tanno, Paola Giardullo, Elena Porcù, Elena Rampazzo, Luca Persano, Giampietro Viola, Claire Dalmay, Fabrice Lalloué, Arnaud Pothier, Caterina Merla, Mariateresa Mancuso

**Affiliations:** 1 Laboratory of Biomedical Technologies, ENEA CR-Casaccia Via Anguillarese 301, 00123 Rome, Italy; arianna.casciati@enea.it (A.C.); mirella.tanori@enea.it (M.T.); barbara.tanno@enea.it (B.T.); 2 XLIM, University of Limoges, UMR 7252, F-87000 Limoges, France; remi.manczak@xlim.fr (R.M.); claire.dalmay@xlim.fr (C.D.); arnaud.pothier@xlim.fr (A.P.); 3 Captur, University of Limoges, EA3842, F-87000 Limoges, France; sofiane.saada@unilim.fr (S.S.); fabrice.lalloue@unilim.fr (F.L.); 4 Department of Radiation Physics, Guglielmo Marconi University, 00193 Rome, Italy; giardullo_pl@hotmail.it; 5 Department of Women’s and Children’s Health (DSB), University of Padua, 35128 Padua, Italy; elena.porcu@gmail.com (E.P.); elena.rampazzo@unipd.it (E.R.); luca.persano@unipd.it (L.P.); giampietro.viola.1@unipd.it (G.V.); 6 Institute of Pediatric Research Institute (IRP), 35129 Padua, Italy

**Keywords:** D283Med, cancer stem cell, stemness biomarkers, CD133, dielectrophoresis, cross-over frequency

## Abstract

Medulloblastoma (MB) is the most common malignant pediatric brain tumor. Despite the progress of new treatments, the risk of recurrence, morbidity, and death remains significant and the long-term adverse effects in survivors are substantial. The fraction of cancer stem-like cells (CSCs) because of their self-renewal ability and multi-lineage differentiation potential is critical for tumor initiation, growth, and resistance to therapies. For the development of new CSC-targeted therapies, further in-depth studies are needed using enriched and stable MB-CSCs populations. This work, aimed at identifying the amount of CSCs in three available human cell lines (DAOY, D341, and D283), describes different approaches based on the expression of stemness markers. First, we explored potential differences in gene and protein expression patterns of specific stem cell markers. Then, in order to identify and discriminate undifferentiated from differentiated cells, MB cells were characterized using a physical characterization method based on a high-frequency dielectrophoresis approach. Finally, we compared their tumorigenic potential in vivo, through engrafting in nude mice. Concordantly, our findings identified the D283 human cell line as an ideal model of CSCs, providing important evidence on the use of a commercial human MB cell line for the development of new strategic CSC-targeting therapies.

## 1. Introduction

Around 300,000 children and adolescents worldwide are diagnosed with cancer each year [1,2,3,4]. Among pediatric cancers, Medulloblastoma (MB) is the most common intracranial primitive neuroectodermal malignancy, with an incidence of 6 children per million under 9 years of age and less than 2 cases out of a million among 15–19 years of age [5,6]. Despite the current treatment for MB that combines surgery, radiotherapy, and chemotherapy, the risk of recurrence, morbidity, and death still remains highly significant. Extensive genomic analyses have classified MBs into four distinct molecular groups: WNT, SHH, group 3, and group 4 [7,8,9,10,11,12,13,14]. Recent evidence, obtained by single-cell transcriptomics, has shown a huge intertumoral heterogeneity at the molecular level, thus suggesting that MBs should be further subdivided into other molecular subgroups sharing some important deregulated pathways [15].

It has been hypothesized that only a small subset of tumor cells can initiate and support tumor growth. These rare stem cells, called cancer stem cells (CSCs), possess tumorigenic ability with marked capacity for proliferation, self-renewal, and differentiation potential [16,17,18]. In human brain tumors, CSCs were first isolated and characterized in 2004 [19]. Despite in MB, CSCs are present in a very low proportion, and growing evidence shows the importance of their contribution to MB therapy resistance [20,21]. Current radio and chemotherapies efficiently kill the bulk of cancer cells but spare a relevant fraction of CSCs, which are protected by specific resistance mechanisms and peculiar niches in the mass [22,23]. To develop specific CSCs-targeting therapies, studies are needed using enriched and stable CSC cell populations. Given the relatively MB low incidence, the possibility of accessing patient-derived samples is extremely limited and furthermore, few MB cell lines are available in central repositories making it more complex to study this tumor compared to others [24]. Thus, the MB research area is greatly frustrated by the recognized difficulty in culturing and obtaining high amounts of primary patient-derived cells for in vitro studies. Moreover, the use of mice for MB cell propagation is limited by high costs and the management of high-throughput experiments. To validate the stemness lineage of CSCs from MB cells, these can be characterized by the expression of well know stem cell phenotypic markers [25,26,27,28] that are of special interest in understanding the progression of MB [29,30]. Nevertheless, CSCs enrichment often requires particular culture media and presents several difficulties in the experimental protocols, thus resulting in a time-consuming process and suggesting the need for a better characterization of easily available cell lines.

Through a multidisciplinary approach, here we discriminated undifferentiated cell populations in three different MB cell lines (DAOY, SHH group; D341, group 3; D283, group 3/4), as representative of different molecular subtypes [5]. Potential differences in stemness features were analyzed by classical approaches as gene, protein, and flow cytometry analysis of specific stem cell markers. Furthermore, since physical characterization was recently reported as complementary to cell biological features, to identify CSCs, we used high-frequency dielectrophoresis (HF-DEP) crossover frequency, a label-free, accurate, fast, and low-cost diagnostic technique that exploits the polarization and consequent motion of bio-particles in an applied electric fields [31].

## 2. Results

### 2.1. Evaluation of Multiple Stemness Markers

**Figure 1:**
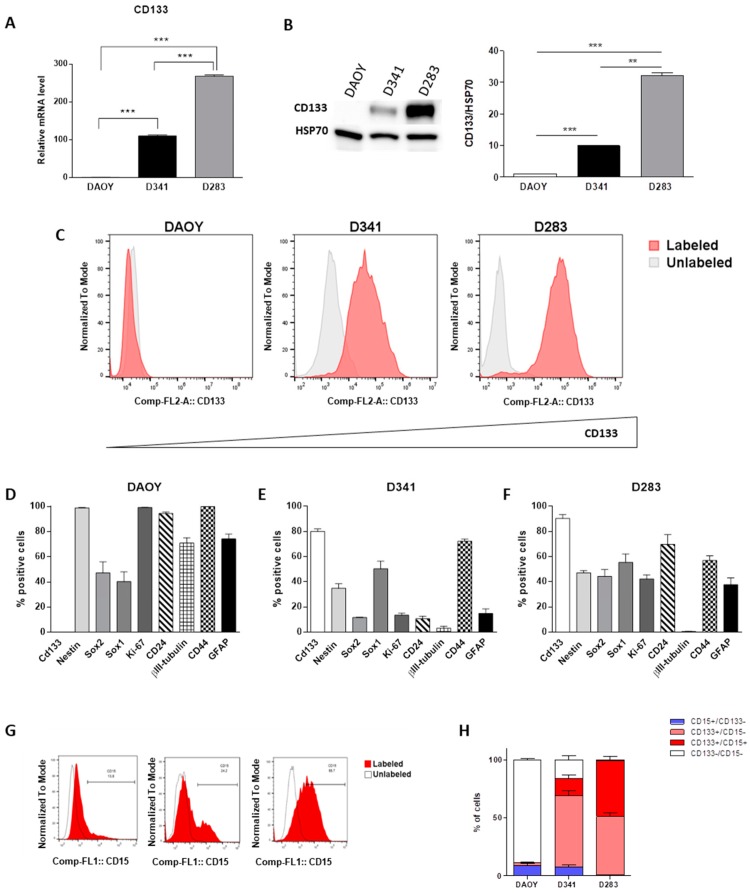
Evaluation of multiple stemness markers in parental medulloblastoma (MB) cells. Gene (**A**) and protein expression of CD133 by Western blot (**B**); band intensities were normalized against HSP70, and DAOY expression level was taken as 1) and flow cytometry analysis. Whole western blots related to main Figure 1B are shown in Fig. S5. (**C**). Stem flow cytometry analysis of DAOY (**D**), D341 (**E**) and D283 (**F**) cells grown in normal medium. Flow cytometry analysis of CD15 expression (**G**) and graphic representation (**H**). Results are expressed as a mean of three biological replicates ± standard error of the mean (SEM). Differences were tested with Student’s t-test. ** *p* < 0.001; *** *p* < 0.0001.

As the first step, we explored potential differences in mRNA expression levels of CD133 in all MB cell lines. Our results showed a significantly higher level of CD133 gene expression in D283 compared with D341 cells and its almost complete lack in the DAOY cell line (Figure 1A). Interestingly, the analysis of protein levels determined by two different techniques, western blot and flow cytometry, showed the same significant trend (Figure 1B,C). For an in-depth analysis of their stemness features, MB cell lines were also stained for various markers of stemness/differentiation. In particular, we considered: (i) The expression of CD133, Nestin, SOX1, and SOX2 as indicative of stemness; (ii) Ki67 for proliferative cells; (iii) GFAP and CD44 as astrocytic markers; iv) CD24 and βIII-tubulin as representative of neuronal progenitors or highly neuronal differentiated cells, respectively. Results are shown in Figure 1D–F and in the Supplementary Information file (Table S1). Notably, although the almost complete lack of CD133+ cells (0.13%), DAOY cells showed the highest expression levels of neural stem/progenitor cell and proliferative markers (Nestin 98.86%, Ki67 99.20%,) simultaneously with the significant overexpression of differentiation markers (βIII-tubulin 70.76%, CD24 94.49%, CD44 99.80% and GFAP 74.20%; Figure 1D). Furthermore, we clearly confirmed that the D341 cell line showed a statistically significant lower CD133 protein expression compared to D283 (80.1% vs. 90.5%; *p* = 0.0158), together with clear-cut reduced expression levels of SOX2 (11.74% vs. 44.40%; *p* < 0.006), Nestin (34.97% vs. 47.24%; *p* < 0.0053) and Ki67 (13.64% vs. 42.27%; *p* = 0.0007). Remarkably, they showed an increased level of CD44 differentiation surface marker, (72% vs. 57.03%; *p* = 0.04; Figure 1E,F). As further support of this evidence, both D283 and D341 cell lines displayed an almost complete lack of βIII-tubulin (respectively, 0.61% and 3.33% vs. 70.96% with *p* < 0.0001 for both comparisons), reduced expression of CD44 (respectively, 57.03% and 72.4% vs. 99.8% with *p* < 0.0001 for both comparisons) and GFAP relative to DAOY cells (37.81% and 14.87% vs. 74.2% with *p* = 0.0011 and *p* = 0.0001, respectively). Phenotypic characterization carried out in this study showed a huge heterogeneity for stemness/differentiation-related markers, especially between D283 and DAOY cells and, importantly, that their expression levels were not influenced by oxygen culture conditions (Figure S1). Interestingly, the analysis of an important CD133 downstream stem cell regulatory gene, such as BMI1, showed a significantly higher expression level in D283 cells with respect to other MB cell lines (Figure S2; *p* < 0.0001). In addition to CD133, we analyzed CD15, which reported a significantly higher percentage of CD15-positive cells in D283 cells (52.5%) than D341 (23.3%) and DAOY (9.3%; Figure 1G,H; Table S1). Of note, almost 50% of D283 cells showed co-expression of CD133 and CD15 (Figure 1H) compared to significantly lower proportions in D341 and DAOY (14.6% and 0.22%, respectively, Figure 1H).

### 2.2. Medullospheres Characterization

As stemness can be measured by the ability to form spheres when cultured in stringent conditions, MB cells were cultured at clonal density in a selective medium for 7 days, in the absence of serum.

**Figure 2:**
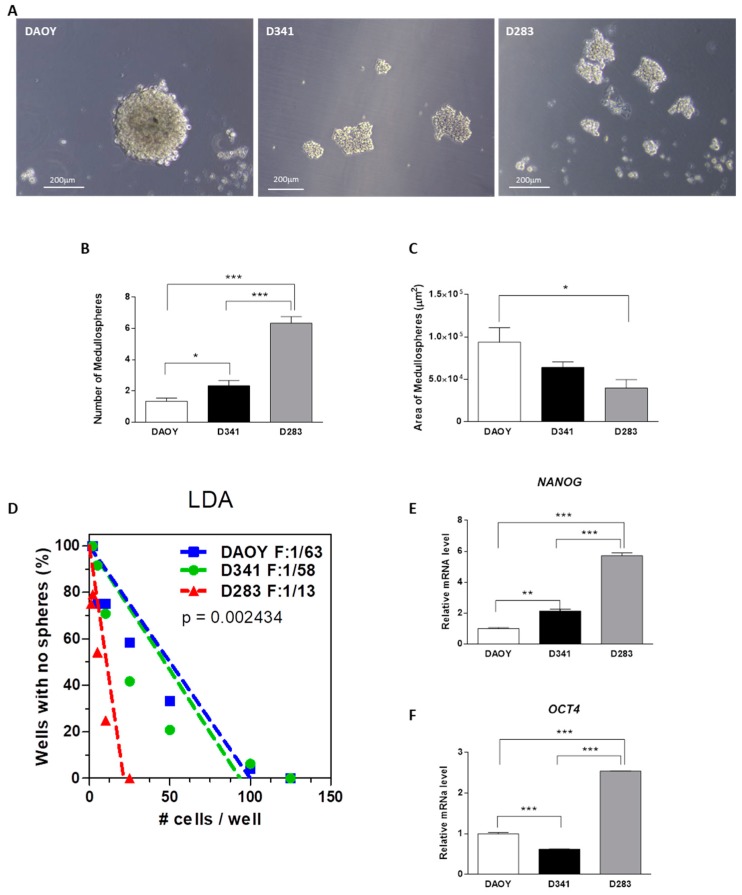
Medullospheres (MS) assay. Representative images of MS obtained with MB cell lines (**A**). MS quantitative analysis: Number (**B**), area (**C**). The fraction of wells without MS plotted against cell numbers per wells (LDA, **D**). Protein expression and relative densitometry of CD133 (**E**) Gene expression of NANOG (**F**) and OCT4 in basal conditions; DAOY expression levels are taken as 1. Data are shown as a mean of three biological replicates ± SEM. Differences were tested with Student’s *t*-test. * *p* < 0.05, ** *p* < 0.001; *** *p* < 0.0001. Bars: 200 μm.

DAOY cells generated an extremely low rate of medullospheres (MS), characterized by large and regular shapes (Figure 2A–C). The number of MS obtained from D341 cells was significantly higher compared with DAOY-MS, but dimensionally we did not observe significant differences (Figure 2A–C). Notably, D283 cells generated the highest number of MS, although they had the smallest size (Figure 2A–C). The statistically significant increase of CD133 at protein level confirms the undifferentiated cell enrichment after MS assay (Figure S3). According to MS generation ability, the limiting dilution assay (LDA) clearly shows a significantly higher frequency of initiating cells (F = 1/13) in D283 than D341 (F = 1/58) and DAOY (F = 1/63) cells (Figure 2D). Finally, to better understand the genetic stemness regulatory network in our cell lines maintained in basal culture conditions, we carried out the analysis of two essential transcription factors (NANOG and OCT4) that regulate self-renewal and pluripotency of stem cells. Our results showed a significant increase in gene expression in D283 cells with respect to other MB cell lines (Figure 2E,F; *p* < 0.0001), strongly highlighting a cancer stem-like phenotype of D283 cells.

### 2.3. HF–DEP Crossover Frequency

**Figure 3:**
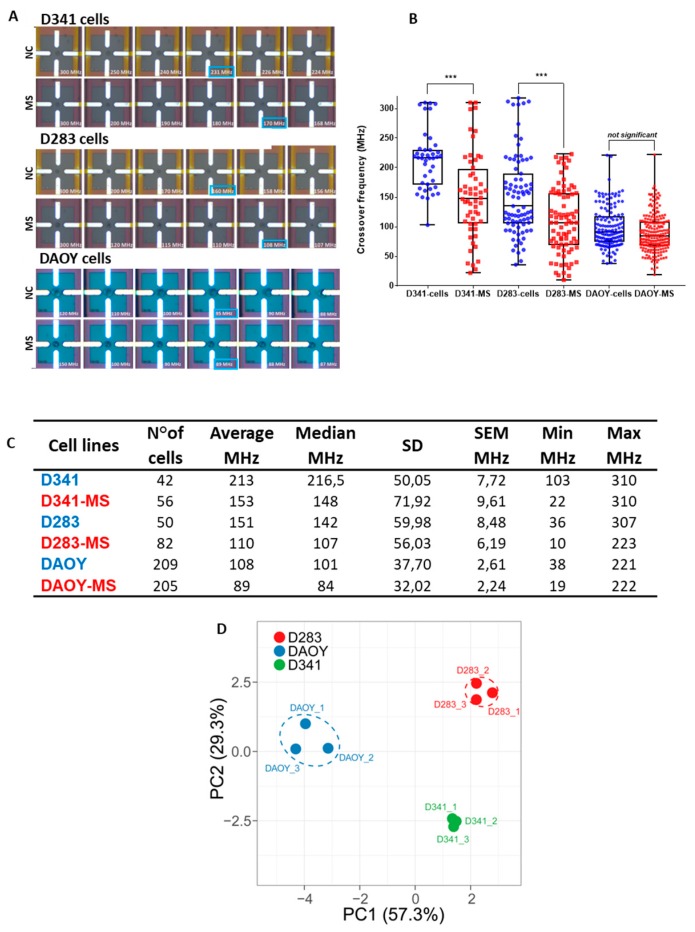
Crossover frequencies of MB cells. Graphic box plots representation of D341 and D283 cells crossover frequencies, cultured in two different conditions: Normal medium (NC) and MS medium (**A**,**B**). The middle bar represents the median value of the collected data. Summary of the statistic parameters concerning the characterization of crossover (**C**). The *p*-value was determined using a one-way ANOVA test. Differences in crossover frequencies obtained for each subpopulation show a *p*-value = 2 × 10^−5^. *** *p* < 0.0001. Graphic representation of the Principal Component Analysis (PCA) generated by unit variance scaling of data and SVD calculation of components through the ClusVis web tool (**D**).

MB cell lines, cultured in normal or MS medium, were also characterized by establishing their HF–DEP crossover frequency, defined as the value able to move cells from a negative to positive DEP state. The imaging sequences (boxed in blue, Figure 3A and Figure S4) represent the cell trapped by repulsive forces in negative DEP, and then, the cell attraction on the electrode surface in positive DEP after applying a gradual frequency decrease. Considering the measured crossover frequencies, D341-MS and D283-MS showed a statistically significant lower crossover frequency than cells cultured in normal conditions (Figure 3B; *p* = 2 × 10^−5^). On the contrary, DAOY-MS showed very similar crossover frequencies compared with their parental counterpart. The box plots graphic representation illustrates the median together with the min/max crossover frequency for each cell population (Figure 3B). The complete set of statistics concerning the characterization of MB cells crossover frequencies is summarized in Figure 3C, listing the average, median, standard deviation, standard error, and minimum and maximum crossover frequency values for each cell population. Finally, the integration of the several stem cell properties that we analyzed for MB cells through the Principal Component Analysis (PCA) clearly demonstrated that D283, D341, and DAOY cells were characterized by peculiar phenotypic stem cell features, which reflected well their endowed functional ability to generate MS and/or self-renewal (Figure 3D).

### 2.4. Tumor-Propagating Capacity

**Figure 4:**
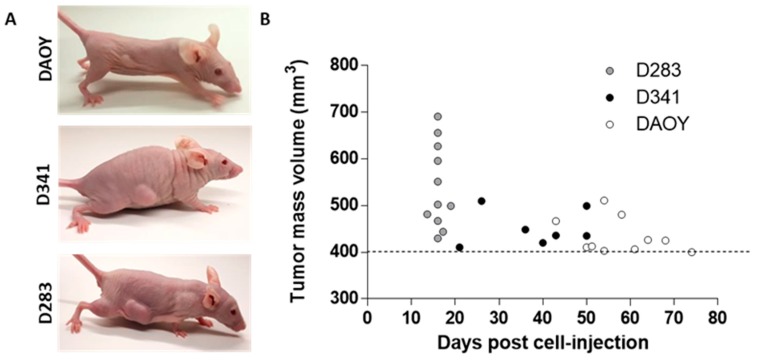
Tumor-propagating capacity. Representative pictures of tumor masses with different dimension at 16 days post-implantation (**A**). Time of appearance of tumors with a volume of ≥400 mm^3^ (**B**).

To examine the tumorigenic potential of MB cell lines, 6 × 10^6^ cells of each cell line was injected subcutaneously into nude mice. Although the subcutaneous injection of brain tumor cells could present some limitations compared to their orthotopic implantation, it ensured quick and easy monitoring of tumor engraftment and progression, and it is generally accepted as a proof of concept of cell tumorigenicity. As shown in Figure 4A,B, D283 cells disclosed 100% of tumor engraftment within 16 days post injection. Tumor incidence, 50 days post-implantation, was 100% (7/7) and 30% (3/10) for D341 and DAOY cell lines, respectively; subcutaneous implantation of DAOY cells reached 100% of tumor incidence within 74 days (Figure 4B). The higher tumorigenic potential shown by D283 cells well reflects the in vitro results demonstrating, concordantly, a cancer stem-like phenotype of D283 cells.

## 3. Discussion 

MB heterogeneity is characterized by the presence of a small population of CSCs, representing the most undifferentiated state of malignant cells with distinct biological characteristics, well-recognized as responsible for relapse and high mortality [32]. Similarly to embryonic and adult stem cells, CSCs express markers that are not expressed in normal differentiated somatic cells and are thus thought to contribute towards a ‘stemness’ phenotype [33]. By analyzing the expression of a broad panel of CSC markers, here we characterized the most frequently used MB cell lines: DAOY (for subgroup SHH), D283-Med (for subgroup 3/4), and D341-Med (for subgroup 3), as representative of patient molecular subtypes [30,34,35]. In particular, we demonstrate that D283 cells exhibit the highest level of CD133 protein expression and a significantly higher expression of CD15, a marker related to high tumor-propagating capacity in a Shh-dependent MB mouse model and also expressed in a subset of human MBs with poor prognosis [26,36]. In addition, D283 cells present very low levels of neuronal and astrocytes differentiation markers such as βIII-Tubulin and GFAP, respectively. Conversely, DAOY cells show an almost complete lack of CD133 protein expression, a lower level of CD15, although they display a significant amount of some stemness and proliferative markers (such as Nestin, Ki67, CD24). D341 cells display an intermediate phenotype, showing almost 80% of CD133 positive cells but intermediate levels of other stem cell markers. A possible explanation for this heterogeneity could depend on the cell of origin of these MB cell lines. For example, it was reported that DAOYs are enriched in progenitor cells expressing Nestin committed to the granule neuron lineage that exhibits more severe genomic instability and gives rise to tumors more efficiently than conventional granule neuron precursors [37]. These data suggest that the DAOY tumorigenic potential is due to SHH deregulation, also responsible for the higher level of Ki67 protein observed in this cell line [17], rather than the presence of the high level of CSCs positive for CD133. On the other hand, the expression of higher levels of Nestin (related to the structural remodeling function) and Ki67 (related to the proliferation process) expression observed in DAOY cells could point out a complex role in regulation of cell remodeling rather than a stemness feature [38].

CD44 and CD24 have been widely analyzed in combination with other stem cell markers to isolate CSCs from a solid tumor [39,40,41]. When analyzed in MB cells, we found a high variability of their expression among cell lines, with no correlation with CD133 expression level. This ambiguous result is in line with many studies showing great variation of expression of these proteins in cell lines and even in cells of the same cancer subtype, raising a question of reliability regarding their use as CSC surface markers [42,43], in conjunction with its common use as an astrocytic differentiation marker in normal neural cells [44]. In agreement, we found that D283 cells showed the lowest CD44 expression level compared with D341 and DAOY cells. Of note, the opposite expression level of CD44 and CD24 between D341 (CD44-high/CD24-very low) and D283 (CD44 low/CD24 high) is another important aspect that can explain the difference we found in clonogenicity and tumorigenic potential of these MB cell lines. In fact, it has been demonstrated that an unbalanced expression of these markers (i.e., CD44^+^CD24^−/low^) identify CSCs with distinct levels of differentiation [45]. Remarkably, DAOY and D341 cells also express a significantly higher level of differentiation markers such as βIII-tubulin, confirming a more differentiated state of both MB cells with respect to D283 cells. Interestingly, our results on the self-renewal ability of cells through clonal analysis, demonstrated that the capability to form MS is directly correlated to CD133 expression (D283>D341>DAOY), as already reported by other authors [30,46]. This turns out in a significantly higher level of main transcription factors as BMI1, NANOG, and OCT4, all involved in the gene regulatory networks controlling stem cell properties [47,48,49,50]. In agreement with these data, when engrafted in vivo, D283 cells give rise to tumors with high efficiency. This result well reflects data from the in vitro limiting dilution assay that estimate an approximately 5-fold higher frequency of tumor-initiating cells. Notably, although the proliferation index of D283 is significantly lower compared with DAOY cells, as determined by Ki67 expression, the time of engraftment was extremely short and strictly dependent on the higher CD133 expression. Very recently, CD133-enriched cell population group 3 MB cells were more prone to form tumorspheres and more tumorigenic when compared to CD133-depleted cells, as demonstrated after in vitro and in vivo LDA [51]. Similar features (i.e., high proliferative activity, high colony formation efficiency, enhanced ability to generate tumorspheres enriched in CD133+ cells, as well as higher tumorigenicity in vivo) have been demonstrated in USP-13-Med cells, a new MB cell line with a profile more consistent with that of group 4 tumors, when compared to the DAOY cells [24].

Although the physiological response to external stimuli, given by culture media enrichment, remains an important tool to increase the percentage of CD133+ cells, the novelty of our study consists in the identification of a commercial ready-to-use cell line for improving the study of CSC biology of a tumor with a high risk of mortality. Although the functional role and signaling mechanisms of CD133 are not well understood, the high expression of CD133 is associated with resistance of CSCs to chemotherapeutic agents [52,53,54] and with the high capability in initiating tumors [28,55]. As CD133-positive cells maintain a higher self-renewal capability and multipotency [55], these cells should be the primary target to test the efficacy of new therapeutic agents against MB. Interestingly, a previous report clearly showed that CD133+ MB cells may be more sensitive to the inhibition of peculiar over-activated signaling pathways such as PI3K/Akt/mTOR [56].

Furthermore, D283 cells showed a significantly higher level of CD133+/CD15+, making them more useful to study signals transduction pathways generated from both membrane receptors and to develop new therapeutic approaches compared to DAOY cells, expressing in basal conditions low levels of double-positive cells [57,58].

Importantly, we exploited a novel method to discriminate the cell differentiation status using real-time measurements in a microfluidic lab-on-chip (LOC) platform implemented in CMOS technology [31,59,60]. This method was proven to be an efficient approach to identify circulating tumor cells [61] and physiological cell changes [62]. Notably, differentiated cells can be identified and separated from an undifferentiated subpopulation, on the basis of their different dielectric signature, which determines different crossover frequencies [31,63]. The ability to discriminate CSCs through this technique has been recently proved on CSCs enriched and non-enriched populations of two different glioblastoma cell lines (U87 and LN18), showing differences in the intracellular dielectric characteristics [31]. In this study, we showed that D341- and D283-MS present much lower crossover frequencies than cells cultured in normal conditions, while DAOY-MS show very similar crossover frequencies compared with their parental counterpart, confirming the possibility to apply this novel label-free method to rapidly characterize and identify different CSCs independently from tumor model. This finding suggests a strong correlation between the intracellular dielectric characteristics and CSC-CD133+ cell populations. Indeed, CD133 is a transmembrane protein with an intracellular cytoplasmic tail interacting with distinct cytoplasmic partners altering various cellular functions [64]. Consequently, this may explain changes in crossover frequency that are mainly dependent on cytoplasmic features [59,60].

## 4. Materials and Methods

### 4.1. Cell Cultures

Human MB cell lines (DAOY HTB-186, D341 Med-18 HTB-187, and D283 Med HTB-185) were obtained from American Type Culture Collection (ATCC; Manassas, VA, USA). Cell lines were routinely maintained in complete growth medium Eagle’s Minimum Essential Medium (MEM) with 2 mM glutamine and 100 U penicillin/0.1 mg/mL streptomycin. The DAOY and D283 medium were supplemented with 10% fetal bovine serum, while the D341 line was maintained in complete growth medium MEM with 20% fetal bovine serum. Cells were cultured in standard CO_2_ incubators unless they were exposed to hypoxia in an H35 hypoxic chamber (Don Whitley Scientific Ltd, Shipley, UK) in an atmosphere of 2% oxygen, 5% carbon dioxide, and balanced nitrogen.

### 4.2. Medullosphere Formation Assay

To evaluate the capacity to form MS, cells were plated at clonal density (1–2 cells/mm2) into ultra-low attachment 24-well plates in selective medium (DMEM/F12 supplemented with 0.6% glucose, 25 mg/mL insulin, 60 mg/mL N-acetyl-L-cysteine, 2 mg/mL, heparin, 20 ng/mL EGF, 20 ng/mL bFGF, penicillin-streptomycin and B27 supplement without vitamin A). After 7 days in culture, MS size and number were evaluated.

### 4.3. Limiting Dilution Assay

To assess the initiating cell frequency of MB cell lines, cells were cultured in standard conditions until ready for passaging and then seeded serial dilutions of cells ranging from 1 to 250 cells/well in ultra-low attachment 96 well plates (Corning, New York, NY, USA). Cells were then cultured in selective medium (DMEM/F12 supplemented with 0.6% glucose, 25 mg/mL insulin, 60 mg/mL N-acetyl-L-cysteine, 2 mg/mL, heparin, 20 ng/mL EGF, 20 ng/mL bFGF, penicillin-streptomycin, and B27 supplement without vitamin A) for one additional week. The proportion of wells in which sphere formation was not observed was measured and initiating cell frequency (F) calculated according to the Poisson distribution.

### 4.4. RNA Isolation and Real-Time qPCR (qRTPCR)

RNA isolation from cells was performed with RNeasy Mini Kit (# 74104; QIAGEN, Milan, Italy). After quantification, 2μg of total RNA was reverse transcribed with a high-capacity cDNA Reverse Transcription Kit (Applied Biosystems, Foster City, CA, USA), and qPCR was carried out as previously described [65] Oligonucleotide primers used for quantitative RT-PCR are listed in the Supplementary Information file (Table S2). Reactions were performed in triplicate from each biological replicate. Relative gene expression was quantified using Glyceraldehyde-3-phosphate dehydrogenase (GADPH) as house-keeping gene. The ΔΔCt quantitative method was used to normalize expression of the reference gene and to calculate the relative expression levels of target genes.

### 4.5. Western Blot

Cells were lysed as previously described [65] and immunoblotted using standard procedures. Membranes were incubated overnight at + 4 °C with primary antibodies against CD133 (AC133, Miltenyi Biotec, Bergisch Gladbach, DE) and HSP70 (H5147, Sigma-Aldrich, St. Louis, MO, USA). HRP-conjugated secondary antisera (Santa Cruz Biotechnology) were used, followed by enhanced chemiluminescence (ECL Amersham, Amersham, UK). Immunoreactive bands were visualized using Amersham ECL Prime WB detection reagent (GE Healthcare Europe, Milan, Italy). Images were acquired using an Image 6 quant LAS 500 (GE Healthcare Europe), and densitometric analysis was performed using ImageJ software.

### 4.6. Flow Cytometry Analysis

MB cells were characterized for their expression of stemness/differentiation markers through the BD Stemflow™ Human Neural Lineage Analysis Kit (BD Biosciences, Franklin Lakes, NJ; cat n° 561526), although with some modifications [66]. In particular, suggested antibody combinations were modified in order to include the simultaneous analyses of: PE mouse anti-CD133/1 (AC133) (2μl/10^6^ cells; Miltenyi Biotec, Bergisch Gladbach, DE); Alexa Fluor 647 mouse anti-Nestin, Alexa Fluor 488 mouse anti-Ki-67, PerCP-Cy5.5 mouse anti-Sox1, PerCP-Cy5.5 mouse anti-Sox2, FITC mouse anti-CD44, Alexa Fluor 488 mouse anti-β-Tubulin Class III (Clone TUJ1; BD Biosciences, cat n°560381), Alexa Fluor 647 mouse anti-GFAP and PE-Cy5 mouse anti-CD24 (Beckman Coulter, Brea, CA; cat n°IM2645) and mouse anti-CD15-FITC clone MMA BD Biosciences, cat n° 332778), all used at 4µl/10^6^ cells. Briefly, MBs cell lines were harvested, fixed in BD Cytofix Fixation Buffer, and permeabilized with BD Phosflow Perm Buffer III (BD Biosciences, Franklin Lakes, NJ) according to the manufacturer’s instructions of Stemflow human Neural lineage kit. Samples were stored in Perm Buffer at −20 °C for at least 30 min. After recovering, cells were stained with the above-described antibodies for 30 minutes. Samples were analyzed by a CytoFLEX flow cytometer (Beckman Coulter, Brea, CA). Data are presented as the percentage of positive cells in the live-gated cell population determined by physical parameters.

### 4.7. HF–DEP Crossover Frequency

The HF–DEP crossover frequency is defined as the value able to move cells from negative to a positive DEP state [31,63]. In the Supplementary Information file (Figure S4) the typical DEP cell signature observed at HF associated with the trapped cell location on the sensor is illustrated. As shown, a quadrupole microelectrode sensor implemented in a microfluidic channel was used to allow individual cell electromanipulation and selective DEP characterization. HF signals, ranging from 50 to 350 MHz (at steps of 1MHz), were used to probe the dielectric characteristics of the cell cytoplasm bypassing the plasma membrane and offering unique capabilities to specifically investigate the intracellular cell signatures. According to the applied frequency, one cell can then be individually electromanipulated by negative DEP force (repealing it in the center of the quadrupole electrodes) or by positive DEP force (attracting it on the edge of one of the lateral electrodes). Assessing the HF crossover frequency hence allows discriminating cells by measuring a dielectric specificity of their overall cytoplasm content.

### 4.8. Principal Component Analysis (PCA)

Integration of stem cell properties displayed by MB cell lines was generated by applying PCA to the following parameters obtained from 3 independent experimental replicates: Percentage of CD133, CD15, Sox2, Ki67, Nestin, CD24, βIII-tubulin, Sox1, CD44, and GFAP positive cells, cross-over frequency (MHz), number and area of neurospheres, neurosphere forming ability (%) and initiating cell frequency (%). The graph was generated by unit variance scaling of data and SVD calculation of components through the ClusVis web tool (https://biit.cs.ut.ee/clustvis) [67]. Ellipses represent the prediction that a new observation will fall inside them with the probability of 80%.

### 4.9. Subcutaneous Xenograft Model 

Female nu/nu CD1 mice (n = 14) of 6–8 weeks of age, were purchased from Charles River Laboratories Italia (Lecco, Italy), and housed in sterilized filter-topped cages kept in laminar flow isolators, fed with autoclaved food and water ad libitum, and maintained in 12 h light/dark cycle. Aliquots of 6 × 10^6^ cells (n = 10 per DAOY, n = 10 per D283 and n = 7 per D341) suspended in 200 µL of Matrigel (BD Biosciences) were injected subcutaneously into mice (double or single flank). Inoculated animals were monitored daily and tumors measured with a caliper twice a week. Tumor dimension was estimated using the formula: Tumor volume = length x width^2^/2 and considered positive when tumor mass was ≥ 400 mm^3^. This animal study was performed according to the European Community Council Directive 2010/63/EU, approved by the local Ethical Committee for Animal Experiments of the ENEA, and authorized by the Italian Ministry of Health (n° 80/2017-PR).

### 4.10. Statistical Analysis

All quantitative data were presented as mean ± SD and statistical significance (*p*) was calculated by two-tailed Student’s *t*-test. The crossover frequency data were analyzed using a one-way ANOVA test. Final tumor incidence was determined using Fisher’s exact test. All analyses were carried out using GraphPad Software, San Diego, CA, USA.

## 5. Conclusions

Altogether, our results provide evidence that D283 cells, characterized by an intrinsic ubiquitous expression of CD133, are endowed with stem-like features, are extremely tumorigenic, and characterized by peculiar dielectric characteristics. These features are valuable for designing biologically relevant experimental models in clinically oriented studies, making this cell line instrumental for the study of CSCs biology and for the development of more effective therapies against MB.

## Supplementary Materials

The following supplementary information is available online at https://www.mdpi.com/2072-6694/12/1/226/s1, Table S1: Stemflow human neural lineage analysis in MB cell lines, Table S2: List of primers used for quantitative real-time PCR, Figure S1: Stemflow human neural lineage analysis in different oxygen conditions; Figure S2: Gene expression of BMI 1, Figure S3: CD133 protein expression; Figure S4: HF-DEP crossover frequency, Figure S5: Whole western blots related to main Figure 1.
