# Supplementary material for: Human Medulloblastoma Cell Lines: Investigating on Cancer Stem Cell-Like Phenotype
Source: Cancers (Basel). 2020 Jan 17;12(1):226. doi: 10.3390/cancers12010226 (PMC7016648; doi:10.3390/cancers12010226)
Supplement: Supplementary file 1 [file cancers-12-00226-s001.pdf]

Supplementary material

Human medulloblastoma cell lines: investigating on cancer stem cell-like phenotype

Arianna Casciati[1#], Mirella Tanori[1#], Rémi Manczak[2], Sofiane Saada[3], Barbara Tanno[1], Paola Giardullo[4], Elena Porcù[5], Elena Rampazzo[5], Luca Persano[5,6], Giampietro Viola[5,6], Claire Dalmay[2], Fabrice Lalloué[3], Arnaud Pothier[2], Caterina Merla[1*] and Mariateresa Mancuso[1*]

Supplementary Table S1

Table S1. Stemflow Human Neural Lineage Analysis in MB cell lines.

MARKERS		DAOY (%mean ± SEM)	D341 (%mean ± SEM)	D283 (%mean ± SEM)
STEMNESS	CD133	0,13 ± 0,13	80,11 ± 2,03	90,55 ± 2,90
	CD15	9.300 ± 2.267	23.33 ± 0.6766	52.47 ± 2.987
	Nestin	98,86 ± 0,34	34,96 ± 3,46	47,24 ± 1,73
	Sox2	47.21 ± 8.805	11.74 ± 0.1415	44.40 ± 5.362
	Sox1	40,34 ± 7,74	50,45 ± 10,40	55,55 ± 6,63
	Ki67	99,20 ± 0,20	13,64 ± 1,45	42,27 ± 3,25
DIFFERENTIATION	CD24	94,49 ± 1,14	10,78 ± 3,33	69,84 ± 7,71
	βIII-tubulin	70,96 ± 4,18	3,33 ± 1,49	0,61 ± 0,24
	CD44	99,80 ± 0,01	72,40 ± 2,83	57,03 ± 3,70
	GFAP	74,20 ± 3,81	14,87 ± 3,65	37,81 ± 5,28

Supplementary Figure S1

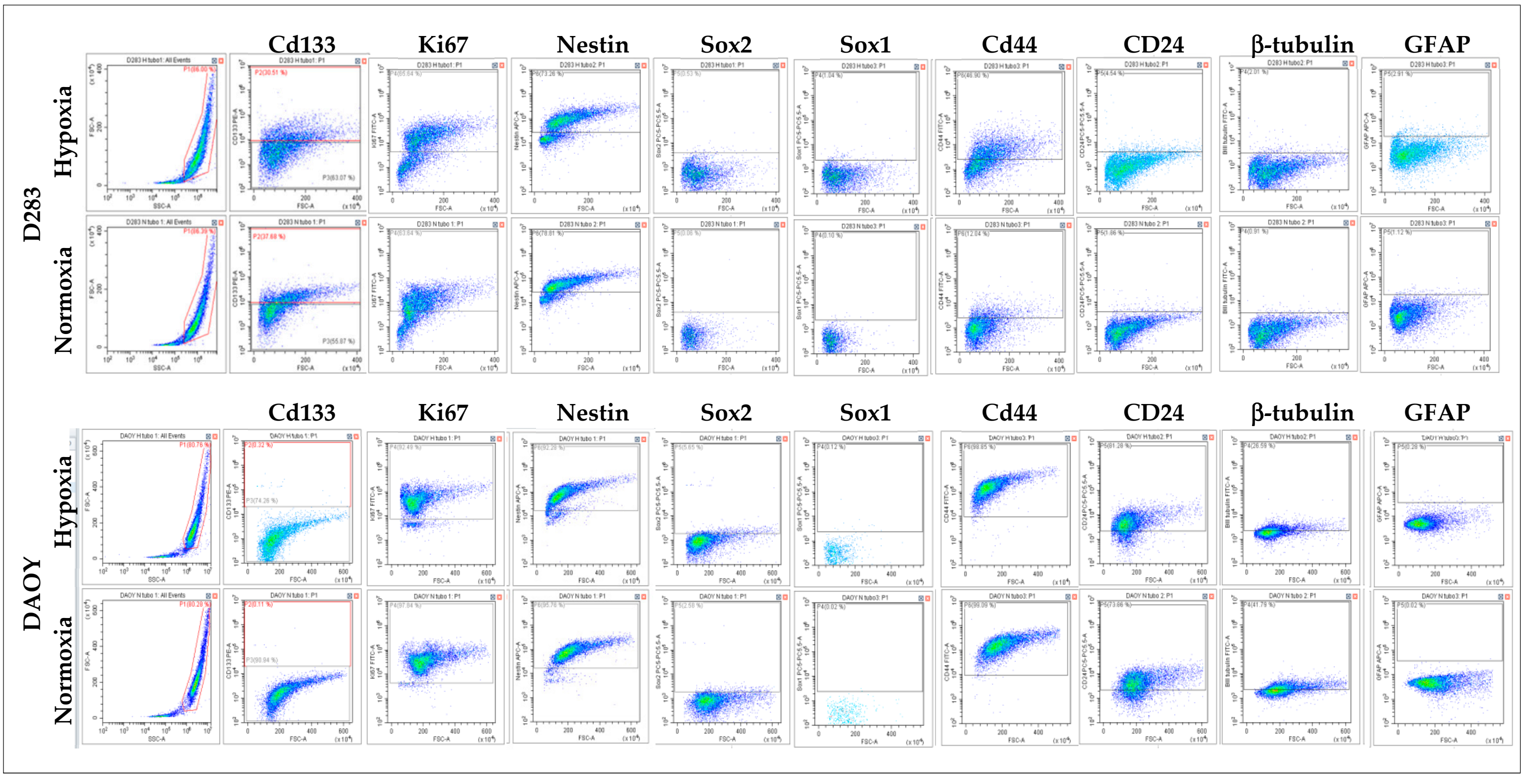

Figure S1. Stemflow Human Neural Lineage Analysis in different oxygen conditions. Representative images of stemflow analysis of D283 and DAOY cell lines showing that their stemness markers expression levels were not influenced by oxygen culture conditions.

Supplementary Figure S2

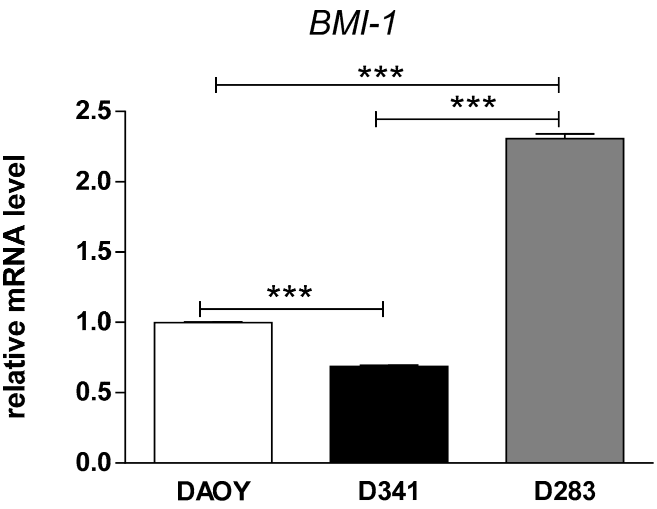

Figure S2. Gene expression of BMI 1. DAOY expression levels are taken as 1. Data are shown as mean of three biological replicates ± SEM. Differences were tested with Student's t-test. *P < 0.05, **P < 0.001; ***P < 0.0001.

Supplementary Figure S3

A

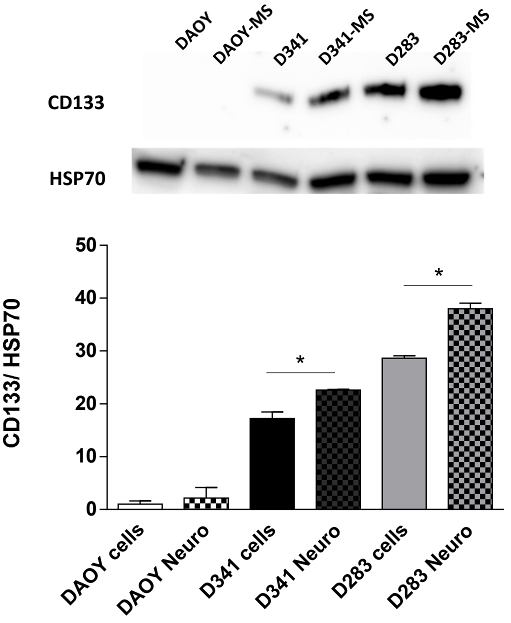

B

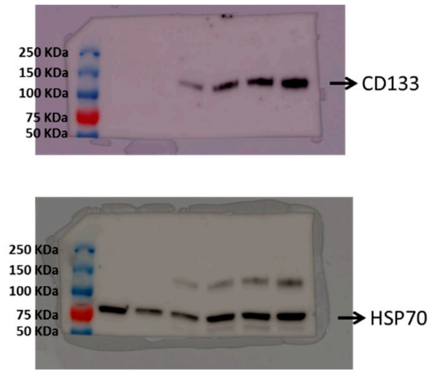

Figure S3. CD133 protein expression. Western blot analysis and relative densitometric representation of CD133 expression level in DAOY, D341 and D283 MB cells, cultured in normal or in MS medium (A). DAOY expression level are taken as 1. Data are shown as mean of three biological replicates ± SEM. Differences were tested with Student's t-test. **P* < 0.05.) Uncropped western blots related to the panel A (B).

Supplementary Figure S4

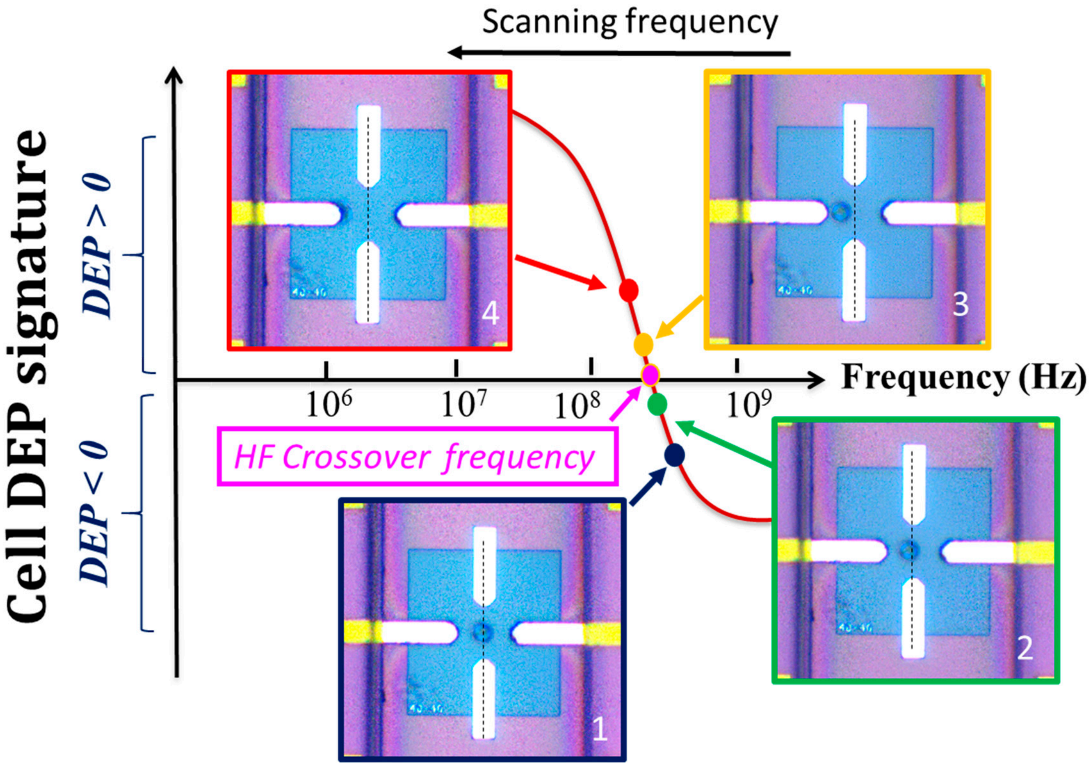

Figure S4. HF cross over frequency. Representative scheme showing cell movement from negative to positive DEP as a function of the applied frequencies (at step of 1 MHz).

Supplementary Table S2. List of primers used for quantitative real time PCR.

Gene	Forward primer	Reverse primer
Cd133	5'- TCCACAGAAATTTACCTACATTGG -3'	5'- CAGCAGAGAGCAGATGACCA -3'
BMI 1	5'- CTTCTGCTGATGCTGCCAAAT-3'	5'- TCCGATCCAATCTGTTCTGG-3'
NANOG	5'- ACCTTGGCTGCCGTCTCTGG -3'	5'- AGCAAAGCCTCCCAATCCCAAACA -3'
OCT4	5'- TTTTGGTACCCCAGGCTATG -3'	5'- TTTTGGTACCCCAGGCTATG -3'
GADPH	5'- ATTCCACCCATGGCAAATTC -3'	5'- GGGATTTCCATTGATGACAAG -3'

Supplementary Figure S5

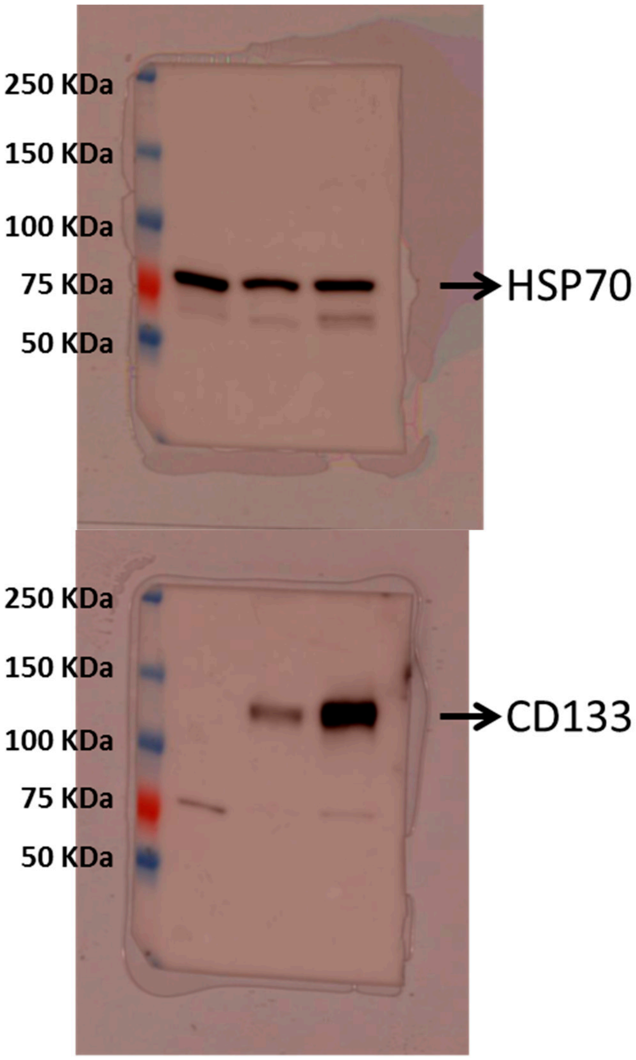

Figure S5. Whole western blots related to main Figure 1.

© 2019 by the authors. Licensee MDPI, Basel, Switzerland. This article is an open access article distributed under the terms and conditions of the Creative Commons Attribution (CC BY) license (http://creativecommons.org/licenses/by/4.0/).
